# Therapeutic efficacy of Sunitinib and other broad spectrum receptor tyrosine kinase inhibitors (RTKI) in bleomycin-induced pulmonary fibrosis

**DOI:** 10.1186/1476-9255-10-S1-P38

**Published:** 2013-08-14

**Authors:** D Knoerzer, T Baginski, K Wade, C Fan, S Rapp, K Regina, F Shih, M Burney, S Rouw, D Welsch

**Affiliations:** 1 Pfizer Global Research & Development, St. Louis Laboratories, Pfizer Inc, St. Louis, MO 63017, USA

## 

Platelet-derived growth factor (PDGF) has ample evidence to support its importance in pulmonary fibrosis. Expression of PDGF in the lungs of IPF patients as well as in BAL samples from animal models of lung fibrosis support its involvement during disease development. Vascular endothelial growth factor (VEGF) being a key regulator of angiogenesis and its receptors have been shown to be involved in the pathogenesis of pulmonary sarcoidosis and fibrosis. The hypothesis under investigation is that receptor tyrosine kinase inhibitors (e.g. Sutent) will slow both the fibrotic processes associated with PDGF signaling and the angiogenic processes regulated by VEGF. We tested two RTKi compounds from Pfizer’s internal portfolio (SU-11248 (Sutent), SU-14813) in the bleomycin-induced pulmonary fibrosis mouse model. Prophylactic as well as therapeutic dosing protocols were conducted. Endpoints included histology, body weights, wet lung weights, lung HO-Pro levels and tissue and serum samples for biomarker and lung exposure analyses. Statistically significant reductions in histological changes were demonstrated in lungs treated with both RTK inhibitors in both percentage of total collagen and inflammatory collagen present. RTK inhibitors demonstrated a statistically significant reduction in both wet lung weight and lung HO-Pro content when dosed prophylactically and therapeutically compared to controls. We have demonstrated that broad-spectrum RTK inhibition is efficacious in inhibiting established pulmonary fibrosis in the bleomycin-induced mouse model. Sutent and other broad spectrum RTKis may provide therapeutic benefit to IPF patients by slowing both the fibrotic and angiogenic processes regulated by tyrosine kinases.

